# Supplementary material for: Loss of centromere function drives karyotype evolution in closely related Malassezia species
Source: eLife. 2020 Jan 20;9:e53944. doi: 10.7554/eLife.53944 (PMC7025860; doi:10.7554/eLife.53944)
Supplement: Figure 1—figure supplement 1—source data 1. [file elife-53944-fig1-figsupp1-data1.docx]

|  | ***M. globosa*** | ***M. slooffiae*** | ***M. furfur*** |
| --- | --- | --- | --- |
| Strain | CBS7966 | CBS7956 | CBS14141 |
| Sequencing platform | PacBio | PacBio | PacBio |
| No. of reads | 122,622 | 92,622 | 57,425 |
| Coverage | 90X | 140X | 64X |
| Size (bp) | 9,119,538 | 8,938,001 | 8,311,773 |
| GC content (%) | 52.09 | 66.31 | 64.9 |
| Assembly status | | | |
| Total contigs | 10 | 14 | 8 |
| Nuclear genome | **Complete**  9 contigs  Telomere on both ends | **Complete**  9 contigs  Telomere on both ends | **Complete**  7 contigs  Telomere on both ends |
| Gaps | None | None | None |
| Assembly polished with Illumina reads | No | No | No |

**Figure 1-figure supplement 1.** Statistics of the genome assemblies of *M. globosa*, *M. slooffiae,* and *M. furfur* generated in this study.
