# Supplementary material for: Loss of centromere function drives karyotype evolution in closely related Malassezia species
Source: eLife. 2020 Jan 20;9:e53944. doi: 10.7554/eLife.53944 (PMC7025860; doi:10.7554/eLife.53944)
Supplement: Figure 2—figure supplement 1—source data 1. — Putative homologs of the indicated proteins in M. sympodialis were identified by BLAST analysis using corresponding protein sequences of C. neoformans as the query. Asterisk (*) indicates cases where corresponding protein sequences from Ustilago maydis were used as query. To detect proteins of the CBF3 complex, protein sequences of Ndc10, Cep3, and Ctf13 from S. cerevisiae were used as query. ‘n.d.’ indicates homolog not detected. [file elife-53944-fig2-figsupp1-data1.docx]

|  | **Sub Complex** | **Query** | **Protein ID**  **(SHOxxxxx.x)** | **% Identity** | **E- value** | **Query coverage** |
| --- | --- | --- | --- | --- | --- | --- |
| **Outer kinetochore** | **Dam1 complex** | **Ask1** | 79565.1 | 27.4 | 4.84e-22 | 65.64 |
|  |  | **Dad1** | 75772.1 | 47.1 | 4.44e-01 | 27.36 |
|  |  | **Dad2*** | 79986.1 | 40 | 9.9e-23 | 82.89 |
|  |  | **Dad3** | 77770.1 | 42.5 | 3.62e-10 | 78.75 |
|  |  | **Dad4** | 79701.1 | 39.1 | 1.24e-09 | 95.83 |
|  |  | **Dam1** | 79667.1 | 43.7 | 6.44e-08 | 45.45 |
|  |  | **Duo1** | 76611.1 | 27.4 | 1.19e-04 | 17.63 |
|  |  | **Spc19** | ND | | | |
|  |  | **Spc34** | ND | | | |
|  |  | **Hsk3*** | 77065.1 | 41.9 | 1.6e-15 | 92.68 |
|  | **Ndc80 complex** | **Ndc80** | 79901.1 | 25.6 | 1.15e-36 | 80.33 |
|  |  | **Nuf2** | 78898.1 | 25.8 | 7.64e-14 | 74.59 |
|  |  | **Spc24** | ND | | | |
|  |  | **Spc25** | 78263.1 | 25.8 | 1.1e-07 | 42.18 |
|  | **Mis12 complex** | **Mis12** | 76526.1 | 25.1 | 5.67e-09 | 70.91 |
|  |  | **Dsn1** | 78762.1 | 24.4 | 3.94e-08 | 24.05 |
|  |  | **Nnf1*** | 77715.1 | 40.8 | 2.10E-19 | 35.9 |
|  |  | **Nsl1*** | 79581.1 | 28.3 | 1.68E-06 | 42 |
|  | **KNL1** | **Spc105** | 75936.1 | 19.9 | 5.36e-11 | 40.59 |
| **Inner kinetochore** | **Constitutive Centromere Associated Network (CCAN)** | **Cnn1/Wip1/**  **Mhf1/Mhf2** | ND | | | |
|  |  | **Mcm16/Mcm22/**  **Ctf3** | ND | | | |
|  |  | **Okp1/Ame1/**  **Ctf19/Mcm21** | ND | | | |
|  |  | **Chl4/Iml3** | ND | | | |
|  | **CENP-C** | **Mif2** | 79930.1 | 35.8 | 5.77e-29 | 22.67 |
|  | **CENP-A** | **Cse4** | 76408.1 | 70.8 | 2.77e-46 | 66.67 |
| **Point CEN specific complex** | **CBF3 complex** | **Ndc10** | ND | | | |
|  |  | **Cep3** | ND | | | |
|  |  | **Ctf13** | ND | | | |

**Figure 2- figure supplement 1. Identification of kinetochore proteins in *M. sympodialis*** **by BLAST.**

Putative homologs of the indicated proteins in *M. sympodialis* were identified by BLAST analysis using corresponding protein sequences of *C. neoformans* as the query. Asterisk (*) indicates cases where corresponding protein sequences from *Ustilago maydis* were used as query. To detect proteins of the CBF3 complex, protein sequences of Ndc10, Cep3, and Ctf13 from *S. cerevisiae* were used as query. ‘n.d.’ indicates homolog not detected.
