# Supplementary material for: Loss of centromere function drives karyotype evolution in closely related Malassezia species
Source: eLife. 2020 Jan 20;9:e53944. doi: 10.7554/eLife.53944 (PMC7025860; doi:10.7554/eLife.53944)
Supplement: Supplementary file 1. [file elife-53944-supp1.docx]

**Loss of centromere function drives karyotype evolution in closely related *Malassezia* species**

Sundar Ram Sankaranarayanan^1^, Giuseppe Ianiri^2,$^, Marco A. Coelho^2^, Md. Hashim Reza^1^, Bhagya C. Thimmappa^1,#^, Promit Ganguly^1^, Rakesh Netha Vadnala^3^, Sheng Sun^2^, Rahul Siddharthan^3^, Christian Tellgren-Roth^4^, Thomas L Dawson Jr.^5,6^, Joseph Heitman^2,*^, and Kaustuv Sanyal^1,*^

**List of primers utilized in this study.**

| **Primers to generate epitope tagging alleles in *M. sympodialis*** | | |
| --- | --- | --- |
| Msy Mtw1 N-P1 | GCGCGCCTAGGCCTCTGCAGGTCGACTCTGACTGACCACGACGAGCTG | Primers to tag Mtw1 with GFP at N- terminus |
| Msy Mtw1 N-P2 | CGCCCTTGCTCACCATCGAGGGGTGGAGGTACAATAG |  |
| Msy Mtw1 N-P3 | CTATTGTACCTCCACCCCTCGATGGTGAGCAAGGGCG |  |
| Msy Mtw1 N-P4 | GCGTCCGAGGTGGACATGTACAGCTCGTCCATGCC |  |
| Msy Mtw1 N-P5 | GGCATGGACGAGCTGTAcATGTCCACCTCGGACGC |  |
| Msy Mtw1 N-P6 | GAGGATCTGCACCGTGGCACATTGCGCGATGATG |  |
| Msy Mtw1 N-P7 | CATCATCGCGCAATGTGCCACGGTGCAGATCCTC |  |
| Msy Mtw1 N-P8 | TGATTACGAATTCTTAATTAAGATATCGAGCGTCCTCTCCTATGTCTGACC |  |
| **Primers to generate epitope tagging alleles in *M. furfur*** | | |
| MfCse4 P1 | GCGCGCCTAGGCCTCTGCAGGTCGACTCTATGCAGCAACAGGCACACATG | Primers to tag CENP-A with 3xFLAG tag at C terminus |
| MfCse4 P2 | CTACTTGTCATCGTCATCCTTGTAGTCGATGTCATGATCTTTATAATCACCGTCATGGTCTTTGTAGTCCCGGATGTCGCCCCAATG |  |
| Mf NAT-F | GACTACAAAGACCATGACGGTGATTATAAAGATCATGACATCGACTACAAGGATGACGATGACAAGTAGTCCACGGTGCAGATCCTCG |  |
| Mf NAT-R | GCTTTCATAGGAACATGCCCTGCGTCCTCTCCTATGTCTG |  |
| MfCse4 P3 | CAGACATAGGAGAGGACGCAGGGCATGTTCCTATGAAAGC |  |
| MfCse4 P4 | TGATTACGAATTCTTAATTAAGATATCGAGGAGGCGATCAACCGGCTTAG |  |
| **Primers for *M. sympodialis* centromeres** | | |
| MS1 F1 | AAGAATTGATAACATTGTTGCAC | *MsyCEN1* primers |
| MS1 R1 | TAGAATAAAATGTCGCGAAGG |  |
| MS2 F1 | CTGAAGAAAAGAAACAAATTCG | *MsyCEN2* primers |
| MS2 R1 | TCGGAAATCCCGCAAAAG |  |
| MS3 F1 | CATATTCAGCCTCCACTAAG | *MsyCEN3* primers |
| MS3 R1 | CCTCTATCGAGTGCTCTAC |  |
| MS4 F1 | CGATATGGATTGGACTTATAAGTC | *MsyCEN4* primers |
| MS4 R1 | AAAAGCAATACGTAGACGG |  |
| MS5 F1 | AAATTACCGACCAGAATTG | *MsyCEN5* primers |
| MS5 R1 | ATCTGTGTCCGCTCTCATC |  |
| MS6 F1 | TTTGACGCTTTATTTGTGTTTC | *MsyCEN6* primers |
| MS6 R1 | CACATATGCACGAATAATAAAACG |  |
| MS7 F1 | GATACATATTCTTACACTAATACTATTCG | *MsyCEN7* primers |
| MS7 R1 | GCATAGAGCTAATATCTGATATTC |  |
| MS8 F1 | GGAAGCATGAGATATTGG | *MsyCEN8* primers |
| MS8 R1 | AAACAAAGTAAAATTCTAATCACG |  |
| MS8 LF1 | CTCCTCCGATACGATTCAC | *MsyCEN8* L1 primers |
| MS8 LR1 | CAGCCATTATCTCCGACAC |  |
| MS8 LF2 | CTGGGTAGATTGAGAATGAG | *MsyCEN8* L2 primers |
| MS8 LR2 | CATGTATGTTCAGTCCCATG |  |
| MS8 RF1 | ATGATCCAAAAGAAAGCATAC | *MsyCEN8* R1 primers |
| MS8 RR1 | GAAGTATGTCTGGGTGAAGC |  |
| MS C3 | GAAGACGACAACGATACC | Control primers away from *MsyCEN1* |
| MS C4 | TAGCGAGTGAATAGCGTC |  |
| **Primers for chromoblot analysis in *M. globosa*** | | |
| Maglo_CBS7966_v2_Chr3_216001_216700_Forward: | GATGAGCGACGGAAACAAGC | Probe for Chr3 |
| Maglo_CBS7966_v2_Chr3_216001_216700_Reverse: | AACTTCGTCCCATTCGCCTT |  |
| Maglo_CBS7966_v2_Chr4_150001_150700_Forward: | CATCGAGATTGCAACACAGC | Probe for Chr4 |
| Maglo_CBS7966_v2_Chr4_150001_150700_Reverse: | TGAACACAGGCGCCATTGTA |  |
| Maglo_CBS7966_v2_Chr5_213001_213600_Forward: | TGCAATGAAGTCCGGCATGA | Probe for Chr5 |
| Maglo_CBS7966_v2_Chr5_213001_213600_Reverse: | AGGCACACGTTCATCTGGTT |  |
| Maglo_CBS7966_v2_Chr6_461001_461600_Forward: | TGCTCACCCAAAAGACGACC | Probe for Chr6 |
| Maglo_CBS7966_v2_Chr6_461001_461600_Reverse: | CGCGGACCTGGAACTGTATT |  |
| **Primers for *M. globosa* centromeres** | | |
| Mg 1F | GAATTGCAATAGTAAGCCGAAC | *MgCEN1* |
| Mg 1R | GAATTATTCAACCCTTTGTACATC |  |
| Mg 2F | GCAAAAGTTCTGGTTAAAC | *MgCEN2* |
| Mg 2R | TTCGTTAAATTACTGTCATTAG |  |
| Mg 3F | GCATGTACAATTCTCTAAAAC | *MgCEN3* |
| Mg 3R | CAAGTTATCTTAATCCGCAAG |  |
| Mg 4F | CAGAAAATAATAGTGATTGATAC | *MgCEN4* |
| Mg 4R | ATTTAAGATACATACACAATGC |  |
| Mg 6F | GGAAATCCTGCGAGAATC | *MgCEN6* |
| Mg 6R | GCTGAATTCATAGAATCATTGAG |  |
| Mg 7F | GATGATCCCAGTAACAACTG | *MgCEN7* |
| Mg 7R | GGTAGAATTGAATTTGTGTTTATC |  |
| Mg 8F | GACTAGCGAATAAATCAATTGAC | *MgCEN8* |
| Mg 8R | TTAACCGTACCGAAAAACC |  |
| Mg 9F | GAAAATAGTGACTGGTGGAC | *MgCEN9* |
| Mg 9R | GATTCTATTGCTATATTGTGCTTC |  |
| Mg 5F | CTAAAAATGAAATTTGGGATAAAAC | *MgCEN5* |
| Mg 5R | AAGCACGATAAAAATCATAGC |  |
| Mg2 L2F | CGTACCTTGTCCAAGAGC | *MgCEN2* L2 primer pair |
| Mg2 L2R | AGATCCATAGGCTTTGAATGC |  |
| Mg2 L1F | ACCTTCGATTCTGTGACAC | *MgCEN2* L1 primer pair |
| Mg2 L1R | TGTTACACACTTTGCTTCGG |  |
| Mg2 R1F | AGGTCCTGACGATGTAATTG | *MgCEN2* R1 primer pair |
| Mg2 R1R | GTTGTTGATGTATGTCGTTCATG |  |
| Mg2 R2F | AGCTATGCGATGTTGTTCTG | *MgCEN2* R2 primer pair |
| Mg2 R2R | CAGACGAGGAACTATTGTGAG |  |
| Mg C5 | GCATAACATACGAGGATGTGC | Primer for control locus |
| Mg C6 | ATAGTGCCTGAATCTGCTG |  |
| **Primers for *M. slooffiae* centromeres** | | |
| Slo1 FP | CAAATGAGCACAAACGTTG | *MslCEN1* |
| Slo1 RP | GGTAATTTACATTTCTTGTG |  |
| Slo2 FP | ACTCAATAATCCAATAGAACC | *MslCEN2* |
| Slo2 RP | GAGAAAACATAAATGGTAGG |  |
| Slo3 FP | AAACCGATTATCAATTCTCAAATG | *MslCEN3* |
| Slo3 RP | GTATCTGATTTGAAAACCTTCG |  |
| Slo4 FP | TTCACGTGTAGCTACTTG | *MslCEN4* |
| Slo4 RP | AAATACAACAAACAACTAAAACG |  |
| Slo5 FP | GAGCTGTGCAAGGTTAG | *MslCEN5* |
| Slo5 RP | GCCAAACAACGATGACG |  |
| Slo6 FP | AATAGATTTGACAACCTTTGC | *MslCEN6* |
| Slo6 RP | TGCACAATTGTAAGAAAGC |  |
| Slo7 FP | AATGCCAGATGATAAACTAGCTG | *MslCEN7* |
| Slo7 RP | GACTTCTGGCATAACTATTGG |  |
| Slo8 FP | TTATGCTATTGTTTGAATCCG | *MslCEN8* |
| Slo8 RP | CTATCATTAAACGGAGAATACTC |  |
| Slo9 FP | GACCTAGCTGTGCTTTTAG | *MslCEN9* |
| Slo9 RP | TTTCAGCAGCTTATTAGGC |  |
| Slo C1 | ACGACAAGCGTGTAAGG | Primers for control locus |
| Slo C2 | CCAACTTCTTCCTGCAG |  |
| Slo1 L2F | GGAACGTGACGAGATCAC | *MslCEN1* L2 primers |
| Slo1 L2R | GTGTAGATCCGAAGTCATCAC |  |
| Slo1 L1F | ATCTCTGCAAGCTTCGG | *MslCEN1* L1 primers |
| Slo1 L1R | AGTGGATGCTTCATCTTCTG |  |
| Slo1 R1F | CGAATGACTTCCTCAATGC | *MslCEN1* R1 primers |
| Slo1 R1R | TGCAACAGCAGAAGAGTC |  |
| Slo1 R2F | ATGCCGACCACAATCC | *MslCEN1* R2 primers |
| Slo1 R2R | ACTGTGCCTGTTTCGC |  |
| **Primers for *M. furfur* centromeres** | | |
| MF1 F1 | GATAGCAAACATGATTAAAGTAATAAC | *MfCEN1* |
| MF1 R1 | GACCAAAATAATATATATTAACAAATG |  |
| MF2 F1 | CAAAAGTGAAGAAGCAGG | *MfCEN2* |
| MF2 R1 | CACATATAAGAAGTAGAAAAGAAAACTC |  |
| MF3 F1 | CATGTCTGGACCTCGG | *MfCEN3* |
| MF3 R1 | CGTGGTGAGAACACAAC |  |
| MF4 F1 | CCTAAACTTATGAACTGTTTATTC | *MfCEN4* |
| MF4 R1 | GTTAAGTATTCCATAATGCTC |  |
| MF5 F1 | CTTCTGCCATCGTTTCTC | *MfCEN5* |
| MF5 R1 | CTTGATTGTTCCTTCGTAATTAAC |  |
| MF6 F2 | CATGTATGTTAAACGTCATAGTAC | *MfCEN6* |
| MF6 R2 | CGATTTGATCTATAATAACATAC |  |
| MF7 F1 | GTGAAGCTATAATATTATAGAATGAG | *MfCEN7* |
| MF7 R1 | CGTTTGAATCATTATAATACTG |  |
| MF7 LF1 | GAAAGCTTCATTCGGAGC | *MfCEN7* L1 primer pair |
| MF7 LR1 | CGTCTTGGGAAGAGCAG |  |
| MF7 LF2 | GGCGGATCATCTTTTCG | *MfCEN7* L2 primer pair |
| MF7 LR2 | GATTCTGATCGTCGGAGG |  |
| MF7 RF1 | GTGGCACTACTGGATCG | *MfCEN7* R1 primer pair |
| MF7 RR1 | CGTGTACCGGTACATGTG |  |
| MF7 RF2 | CTGTACCGCTACCTGC | *MfCEN7* R2 primer pair |
| MF7 RR2 | GTACGAATCGAGATCAACTG |  |
| GI154 | GTCGGAGAAGCAGTCAATGC | Primers for control locus |
| NAT sFP | GTGCGGAGAAGGCATTGTTC |  |
